# Insensitivity versus poor response to tumour necrosis factor inhibitors in rheumatoid arthritis: a retrospective cohort study

**DOI:** 10.1186/s13075-020-2122-5

**Published:** 2020-03-04

**Authors:** Sae Ochi, Kazuyoshi Saito, Fumitaka Mizoguchi, Shigeaki Kato, Yoshiya Tanaka

**Affiliations:** 1 Department of Laboratory Medicine, Jikei University School of Medicine, Nishi-shinbashi 3-25-8, Minatoku, Tokyo, 105-8461 Japan; 2 First Department, University of Occupational and Environmental Health, Iseigaoka 1-1, Yawatanishi-ku, Kitakyushu, Fukuoka, 80708556 Japan; 3 Department of Rheumatology, Graduate School of Medical and Dental Sciences, Tokyo Medical and Dental University (TMDU), Yushima 1-5-45, Bunkyo-ku, Tokyo, 113-8510 Japan; 4 Center for Regional Cooperation, Iwaki Meisei University, Chuodai Iino 5-5-1, Iwaki, Fukushima, 970-8551 Japan

**Keywords:** Rheumatoid arthritis, Tumour necrosis factor inhibitors, Insensitivity

## Abstract

**Background:**

With advancement in the treatment options of rheumatoid arthritis (RA), optimising the outcomes of difficult-to-treat patients has become increasingly important in clinical practice. In particular, insensitivity to first-line biologic disease-modifying anti-rheumatic drugs (bDMARD) is becoming a significant problem because it may decrease the treatment adherence of patients. This study aimed to compare RA patients with an insensitivity and those with a poor response to initial treatment with tumour necrosis factor inhibitors (TNFis), which are the most frequently used bDMARDs.

**Methods:**

This is a retrospective cohort study using clinical data from the FIRST registry. bDMARD-naïve RA patients treated with tumour necrosis factor inhibitors (TNFis) from August 2003 to May 2019 were included and categorised into three groups: TNFi insensitivity, poor response to TNFis and controls. TNFi insensitivity was defined as follows: (1) discontinuation of TNFi treatment within 22 weeks due to lack of any response, or (2) an increase in the disease activity score in 28 joints–C-reactive protein (DAS28-CRP) of > 0.6 at week 22 compared with week 0. Among the remaining patients, those with a DAS28-CRP > 2.6 at week 22 were categorised in the poor response group.

**Results:**

Of the included patients, 94 were classified in the insensitivity, 604 in the poor response and 915 in the control. A higher DAS28-CRP before treatment was a risk factor for a poor response but not for insensitivity. In contrast, dose escalation of infliximab decreased the risk of a poor response but not that of insensitivity.

**Conclusions:**

In future research, poor and insensitivity to bDMARDs should be assessed separately to fully elucidate the aetiology of, and risk factors for, bDMARD refractoriness.

## Background

The development of biologic disease-modifying anti-rheumatic drugs (bDMARDs) has dramatically expanded the therapeutic options for patients with rheumatoid arthritis (RA) who are refractory to conventional synthetic DMARDs (csDMARDs). Global evidence indicates that when RA patients are refractory to csDMARDs, additive bDMARD treatment leads to clinical remission in approximately 30–60% of the patients, a majority of whom also achieve structural remission [1]. Moreover, when treated with a bDMARD during the early stage of the disease, approximately half of RA patients can successfully remain in clinical remission without the need for bDMARDs with no radiological or functional damage progression of articular destruction [2, 3].

Even so, 20–30% of patients with RA remain refractory to treatment [4–6], and only half of patients treated with any single agent have a major benefit [7]. Refractory RA involves a variety of concepts [8]: thus far, the definition of this status is arbitrary, and data on the outcomes of these patients remain limited [9]. From a clinical viewpoint, the refractory status of RA can be roughly categorised into three groups: insensitivity, in which no improvement is observed from the start of treatment; secondary refractory, in which partial, but insufficient, improvement is evident; and false refractory, in which patients complain persistent pain though inflammation is absent [9].

Among these three conditions, identifying insensitivity to bDMARDs has significant clinical importance, because these patients experience no improvement in their symptoms by treatment, thus incurring unnecessary costs and toxicity [10]. Although a second bDMARD is recommended for both intrinsic and secondary refractory RA patients [11], total unresponsiveness might decrease the patient’s motivation to try another treatment. In contrast, partial improvement of symptoms may increase the patient’s adherence to bDMARDs, even if the effect is not sufficient. Therefore, identification of the factors causing insensitivity to bDMARDs is important for avoiding initial failure and improving patient adherence to subsequent therapies.

TNFis are the most frequently used and thus most well-studied bDMARDs. Several factors potentially affecting the response to TNFis have been reported. It has been demonstrated that a high education level [12], low disability grade [13, 14] and usages of nonsteroidal anti-inflammatory drugs [13] and methotrexate (MTX) [15–17] are associated with a good response to TNFis, whereas a young age [18], obesity [19], smoking [20], high disease activity at the time of treatment [13], glucocorticoid usage [12, 18] and positivity for rheumatoid factor (RF) and anti-citrullinated protein (CCP) antibodies [18, 21] are associated with a poor response. However, these findings have not always been consistent across studies [8]. As a result, the choice of treatment usually depends on the route of administration, dose interval, chemical structure and cost rather than etiological factors. Considering the heterogeneity of the status of RA treatment refractoriness, this inconsistency may be due to including different refractory status types in one group. To achieve more personalised treatment, greater knowledge regarding categorisation of the refractory status is needed.

In this study, we retrospectively compared an insensitivity with a poor response to TNFis among bDMARD-naïve RA patients with an inadequate response to csDMARDs (csDMARD-IR). The results may aid future assessments of the effectiveness of bDMARDs and improve optimisation of RA treatments.

## Methods

### FIRST registry

**Fig. 1:**
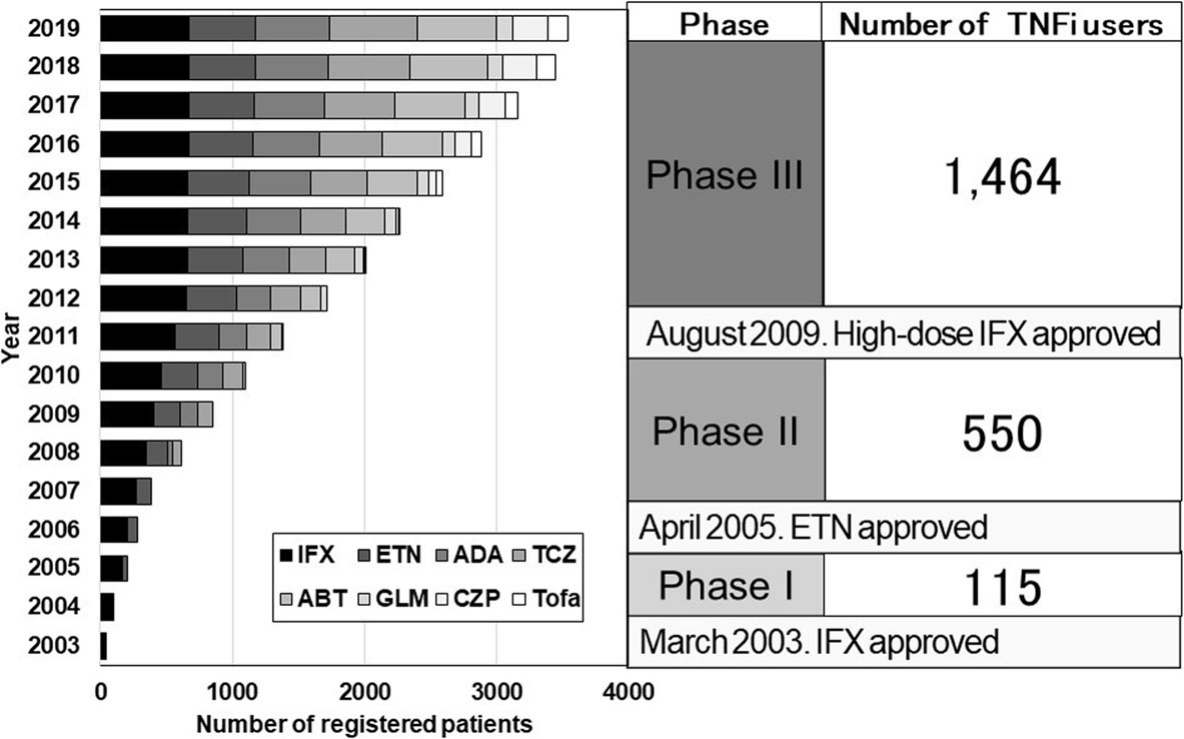
Description of FIRST registry. Number of registered patients and distribution of biological disease-modifying anti-rheumatic drug (bDMARD) users by drug types in each year is shown. IFX, infliximab; ETN, etanercept; TCZ, tocilizumab; ADA, adalimumab; ABT, abatacept; GLM, golimumab; CZP, certolizumab; Tofa, tofacitinib

This was a retrospective cohort study using data from the FIRST registry, a cohort recruited by the University of Occupational and Environmental Health. The hospital and its affiliated hospitals accumulated data from RA patients treated with bDMARDs since the approval of the first DMARD in Japan in 2003 until May 2019. A total of 3389 patients were enrolled in this registry during this period, among whom TNFis (infliximab, etanercept, adalimumab, golimumab and certolizumab) were the most frequently used DMARDs (*n* = 2133) (Fig. 1).

### Study patients

The clinical data of RA patients enrolled in the FIRST registry from August 2003 to May 2019 were collected. Patients with csDMARD-IR and bDMARD-naïve at the time of the treatment with a TNFi were included.

### Definitions of TNFi insensitivity and poor response to TNFis

TNFi insensitivity was defined as either of the following conditions: (i) discontinuation of TNFi treatment within 22 weeks due to a lack of response or (ii) a significant increase in the disease activity score in 28 joints (DAS28)–C-reactive protein (CRP) at week 22 compared with week 0. Some previous studies assessed unresponsiveness at weeks 12–16 [9], but in this study, a proportion of the patients did not have data from that period, and thus week 22 was used as our assessment time point. For condition i above, a lack of response to TNFis was made by the RA specialists treating the patient. For condition ii, the DAS28 may fluctuate to some degree even if disease activity does not change. Therefore, an increase in the DAS28 of > 0.6 was considered meaningful, based on the results of a previous study [22].

A poor response to TNFis was defined as moderate to high disease activity (DAS28-CRP of > 2.6 at 22 weeks or DAS28-ESR of > 3.2). Other patients including those whose treatment was stopped were categorised in the control group.

### The phases of the FIRST registry

The FIRST registry consists of three phases (Fig. 1). Phase I started from the time of approval in Japan of the first TNFi, infliximab, to the approval of the second TNFi, etanercept, at which time phase II began. Therefore, in phase I, RA patients had only one TNFi option: infliximab. When high-dose (10 mg/kg) infliximab was approved, phase III began.

### Statistical analysis

For continuous variables, the normality of the data was assessed using the Shapiro–Wilk test. For categorical variables, differences between groups were assessed using the chi-squared test. Associations of gender, age, disease duration, disease activity, RF, use of MTX, use of glucocorticoid, types of drugs and phases with the insensitivity and poor response groups were calculated using multiple logistic regression analysis.

The data available in this registry were age, disease duration, parameters used for calculating the DAS28 (number of tender joints, number of swollen joints, patient global health, CRP titre and erythrocyte sedimentation rate), doses of glucocorticoid and MTX, usage of other anti-rheumatic drugs, full health assessment questionnaire (HAQ), other laboratory data including levels of RF and anti-citrullinated protein antibodies, matrix metalloproteinase-3 (MMP-3), Krebs von den Lugen-6, bone alkaline phosphate and type I collagen cross-linked N-telopeptide (NTx). As the numbers of swollen joints and tender joints and serum levels of inflammation markers are incorporated in the DAS28 calculation, these variables were not additionally included in the analysis. Other variables strongly interact with each other; for example, the HAQ may confound the DAS28 and levels of MMP-3 and bone alkaline phosphate, while NTx may confound glucocorticoid usage. In addition, due to the relatively small sample size of the TNFi insensitivity group, it seemed inappropriate to use regression model-fitting methods. Therefore, age, DAS28, use of glucocorticoids and MTX and RF positivity were selected as explanatory variables. To determine whether the variables interact with each other, logistic regression was also conducted with terms of interaction. *p* value < 0.05 was considered statistically significant. All analyses were conducted using STATA/SE 13.1 (StataCorp LP, College Station, TX, USA).

## Results

**Fig. 2:**
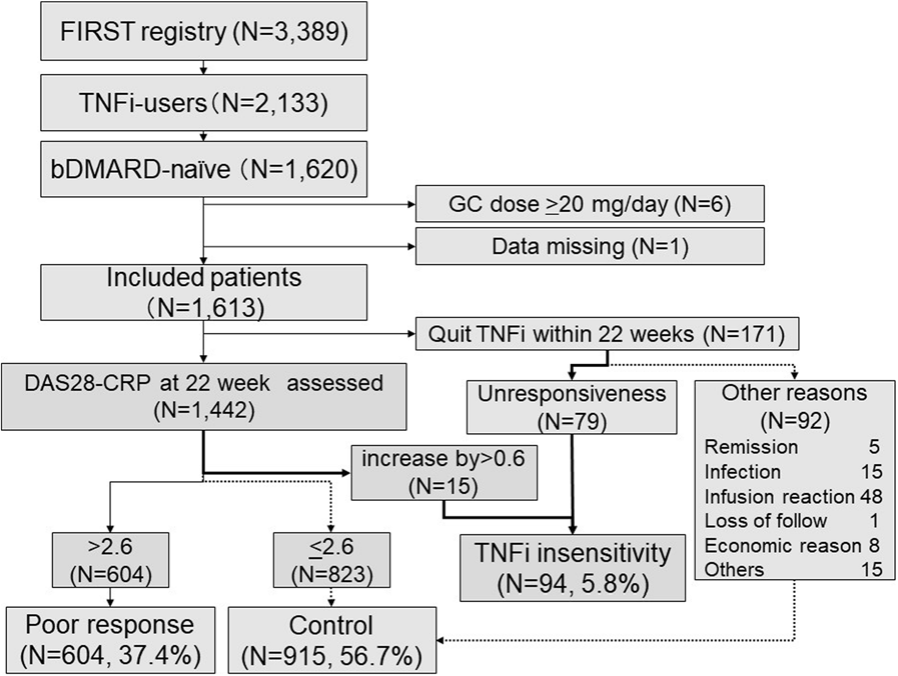
Screening process to identify TNFi insensitive patients. TNFi, tumour necrosis factor inhibitor; bDAMRD, biologic disease-modifying anti-rheumatic drug; DAS, disease activity score, CRP, c-reactive protein

In total, 1620 patients with bDMARD-naïve and csDMARD-IR RA were enrolled. Six patients were excluded because they received a higher dose of glucocorticoids (≥ 20 mg/day) for the treatment of complications such as interstitial pneumonia and other autoimmune diseases. Another patient was also excluded because of missing data about the use of csDMARD. Among the remaining 1613 patients, 172 discontinued TNFi treatment within 22 weeks. Seventy-nine discontinued because of a poor treatment response and 92 discontinued from other reasons such as adverse effects or economic reasons (Fig. 2). Among the remaining 1442 patients, 15 had an increase in the DAS28 of > 0.6. As a result, 94 (6.2%) were classified in the TNFi insensitivity group. Among the remaining, 604 showed DAS28-CRP > 2.6 at week 22 and thus were classified in the poor response group. Those who showed low disease activity at week 22 and those who discontinued the treatment within 22 weeks for reasons other than poor treatment response were allocated to the control group (*N* = 915, Fig. 2).

### Background characteristics

The background characteristics of the included patients are summarised in Table 1. The Shapiro–Wilk test revealed a non-normal distribution for all of the continuous variables listed in Table 1. Therefore, these variables were converted into categorical variables for the statistical analyses. For RF and glucocorticoid, RF positivity and glucocorticoid usage were used instead of RF titre and glucocorticoid dose, respectively. Disease duration, MTX dose and number of treatment failures to previous csDMARDs were categorised.

**Table 1:** Patient background characteristics

Background characteristics		Mean	Median
Age		59.2	61.0
Disease duration (months)		80.2	36.0
Number of swollen joints		8.2	7.0
Number of tender joints		9.4	8.0
DAS28ESR		5.7	5.7
DAS28CRP		5.0	4.9
ESR (mm/h)		51.4	47.5
CRP (mg/dL)		2.6	1.2
RF (IU/mL)		164.9	64.4
MMP-3 (ng/mL)		272.5	152.0
Dose of MTX (mg/week)		9.7	10.0
Dose of GC (mg/day in prednisolone equivalent)		1.3	0.0
Failure in csDMARDs		2.0	2.0
		Frequency	Percent
Gender	Female	1307	81.0
	Male	307	19.0
Entry phase	I	115	7.1
	II	442	27.4
	III	1056	65.5
Biologics	IFX	643	39.8
	ETA	372	23.1
	ADA	404	25.0
	GLM	25	1.6
	CZP	170	10.5
Use of corticosteroid		446	27.6
Use of MTX		1436	89.0
Failure in > 2 csDMARDs		903	56.0
RF positive*		1215	75.6

*RF > 20 IU/mL was defined as positive

*CRP* C-reactive protein, *ESR* erythrocyte sedimentation rate, *DAS28* disease activity score for 28 joints, *RF* rheumatoid factor, *MMP-3* matrix metalloproteinase, *MTX* methotrexate, *csDMARDs* conventional synthetic disease-modifying anti-rheumatic drugs

Comparisons of the background characteristics among the three groups are shown in Table 2. Each variable was compared between the groups using the chi-squared test, which revealed significant differences in disease duration, phases of the FIRST registry and TNFi drug type between the insensitive and poor response groups. Many factors showed significant differences between control and poor response groups, but only disease activity at week 0 showed significant difference between control and insensitivity groups.

**Table 2:** Comparison of patient background characteristics between a TNFi insensitivity group and a poor response group. For each variable, *p* values of a simple comparison between the two groups using chi-squared test are shown

Category		TNIi insensitivity		Refractory		Control		*p* value*		
		*N*	%	*N*	%	*N*	%	P1	P2	P3
Gender	Female	75	79.8	501	82.9	731	79.9	0.83	0.14	0.34
	Male	19	20.2	103	17.1	184	20.1			
Age group	≦50	23	24.5	129	21.4	228	24.9	0.62	0.41	0.48
	51–60	26	27.7	149	24.7	205	22.4			
	61–70	19	20.2	165	27.3	246	26.9			
	71	26	27.7	161	26.7	236	25.8			
Disease duration (year)	< 0.5	22	23.4	75	12.4	165	18.0	0.45	< 0.01^†^	0.01^†^
	0.5–2	31	33.0	163	27.0	284	31.0			
	2–5	17	18.1	117	19.4	159	17.4			
	≥ 5	24	25.5	247	40.9	306	33.4			
DAS28-CRP at week 0	< 3.2	3	3.2	6	1.0	43	4.7	< 0.01^†^	< 0.01^†^	0.18
	3.2–5.1	12	12.8	94	15.6	259	28.3			
	> 5.1	79	84.0	502	83.1	613	67.0			
RF positivity**	Negative	25	26.6	127	21.0	240	26.2	0.95	0.02^†^	0.24
	Positive	69	73.4	473	78.3	673	73.6			
Use of GC	No	66	70.2	393	65.1	708	77.4	0.13	< 0.01^†^	0.30
	Yes	28	29.8	211	34.9	207	22.6			
Dose of MTX (mg/week)	0	13	13.8	81	13.4	83	9.1	0.22	< 0.01^†^	0.47
	1–6	10	10.6	74	12.3	79	8.6			
	7–9	22	23.4	189	31.3	218	23.8			
	10–15	32	34.0	176	29.1	294	32.1			
	> 15	17	18.1	84	13.9	241	26.3			
Failure in > 2 csDMARDs	No	44	46.8	301	49.8	365	39.9	0.16	< 0.01^†^	0.66
	Yes	50	53.2	303	50.2	550	60.1			
Entry phase	I	29	30.9	287	47.5	327	35.7	0.11	< 0.01^†^	< 0.01^†^
	II	24	25.5	169	28.0	179	19.6			
	III	22	23.4	95	15.7	287	31.4			
Drug type	IFX	2	2.1	11	1.8	12	1.3	0.77	< 0.01^†^	< 0.01^†^
	ETN	17	18.1	42	7.0	110	12.0			
	ADA	5	5.3	55	9.1	55	6.0			
	GLM	22	23.4	234	38.7	186	20.3			
	CZP	67	71.3	315	52.2	674	73.7			

*P1, comparison between control and insensitivity; P2, comparison between control and refractory; P3, comparison between insensitivity and refractory

**RF > 20 IU/mL was defined as positive

^†^*p* < 0.05

*DAS28* disease activity score for 28 joints, *CRP* c-reactive protein, *MTX* methotrexate, *IFX* infliximab, *ETA* etanercept, *ADA* adalimumab, *GLM* golimumab, *CZP* certolizumab, *csDMARDs* conventional synthetic disease-modifying anti-rheumatic drugs

### Differences in risk factors between the insensitivity and poor response groups

Logistic regression analysis was conducted to identify factors associated with TNFi insensitivity or a poor response to TNFis, in comparison with the control group (Table 3). As the treatment option of high-dose infliximab became available in phase III of the FIRST registry, the phases were categorised as phase I + II versus phase III.

**Table 3:** Multivariable logistic regression analyses for TNFi-insensitivity and poor response to a TNFi

Category		TNFi insensitivity			Poor response		
		OR	95% CI	*p*	OR	95% CI	*p*
Gender	Female	1 (reference)			1 (reference)		
	Male	0.99	0.57–1.71	0.97	0.77	0.58–1.03	0.08
Age category	≦ 50	1 (reference)			1 (reference)		
	51–60	1.27	0.69–2.34	0.44	1.35	0.97–1.86	0.07
	61–70	0.68	0.35–1.32	0.25	1.14	0.82–1.58	0.43
	≧ 71	0.79	0.40–1.55	0.49	1.04	0.74–1.47	0.83
Duration (year)	< 0.5	1 (reference)			1 (reference)		
	0.5–2	0.87	0.47–1.59	0.65	1.24	0.87–1.76	0.24
	2–5	0.75	0.37–1.53	0.43	1.27	0.86–1.89	0.23
	≥ 5	0.51	0.26–1.01	0.05^†^	1.16	0.81–1.66	0.43
DAS28-CRP	< 2.6	1 (reference)			1 (reference)		
	2.6–4.1	0.52	0.10–2.61	0.42	3.71	0.83–16.53	0.09
	> 4.1	1.46	0.31–6.79	0.63	6.93	1.57–30.48	0.01^†^
RF positivity*		0.99	0.60–1.63	0.97	1.08	0.83–1.41	0.58
Concomitant use of GC		1.71	1.02–2.87	0.04^†^	1.29	1.00–1.67	0.05^†^
Dose of MTX (mg/week)	0	1 (reference)			1 (reference)		
	1–6	1.22	0.45–3.30	0.70	0.86	0.52–1.45	0.58
	7–9	0.88	0.37–2.06	0.76	1.04	0.67–1.60	0.87
	10–15	0.87	0.38–1.97	0.73	0.93	0.60–1.43	0.73
	> 15	0.60	0.24–1.46	0.26	0.69	0.43–1.11	0.13
Failure in > 2 csDMARDs		0.67	0.42–1.07	0.09	0.86	0.68–1.08	0.20
Biologics	IFX	1 (reference)			1 (reference)		
	ETA	1.63	0.77–3.44	0.20	1.19	0.84–1.69	0.33
	ADA	1.03	0.53–2.00	0.94	0.59	0.43–0.83	< 0.01^†^
	GLM	2.19	0.42–11.40	0.35	1.95	0.80–4.77	0.14
	CZP	2.64	1.32–5.28	0.01^†^	0.64	0.41–0.98	0.04^†^
Phase	I + II	1 (reference)			1 (reference)		
	III	1.30	0.71–2.38	0.39	0.65	0.49–0.88	0.01^†^

*DAS28* disease activity score for 28 joints, *CRP* c-reactive protein, *MTX* methotrexate, *GC* glucocorticoid, *OR* odds ratio, *CI* confidence interval

*RF > 20 IU/mL was defined as positive

^†^*p* < 0.05

Sex, age, RF positivity, initial MTX dose and treatment failure to more than two csDMARDs were not associated with the risk of either TNFi insensitivity or a poor response to TNFis. Longer disease duration showed a lower risk of insensitivity. Interestingly, higher disease activity (DAS28-CRP > 4.1) before treatment was associated with a higher risk of a poor response, whereas this association was not observed in the TNFi insensitivity group. By contrast, the risk of a poor response was significantly decreased in phase III compared with phase I + II, suggesting that the higher dose of infliximab improved the responsiveness to TNFis. However, the risk of insensitivity did not change with the registry phase. Treatment with certolizumab showed a higher risk of insensitivity but a lower risk of a poor response. These discrepancies suggest that the two groups are intrinsically different.

We used DAS28-CRP as an indicator of disease activity because titres of ESR were not available for some patients. As DAS28-ESR is a commonly accepted indicator, we also conducted the same analysis with definition that DAS28-ESR > 3.2 as refractory and ≤ 3.2 as control (Table 4). In this case, DAS28-ESR > 3.2 was a risk factor for both insensitivity and refractory status. On the other hand, there was more significant difference in the effect of gender, age category, disease duration, dose of MTX and phase. The effect of using certolizumab disappears.

**Table 4:** Multivariable logistic regression analyses for TNFi-insensitivity and poor response to a TNFi using DAS28-ESR

Category		TNFi insensitivity (*N* = 91)				Insufficient response (*N* = 453)		
		OR	95% CI		*p*	OR	95% CI	*p*
Gender	Female	1 (reference)				1 (reference)		
	Male	0.72	0.40–1.29		0.27	0.49	0.35–0.69	< 0.01^†^
Age category	≦ 50	1 (reference)				1 (reference)		
	51–60	1.26	0.69–2.32		0.45	1.78	1.22–2.60	< 0.01^†^
	61–70	0.72	0.36–1.42		0.34	1.72	1.18–2.51	0.01^†^
	≧ 71	0.99	0.51–1.90		0.97	1.91	1.30–2.81	< 0.01^†^
Duration (year)	< 0.5	1 (reference)				1 (reference)		
	0.5–2	0.37	0.14–0.96		0.04^†^	0.94	0.44–2.01	0.87
	2–5	0.29	0.10–0.84		0.02^†^	0.87	0.40–1.91	0.73
	≥ 5	0.14	0.05	0.41	< 0.01^†^	0.90	0.43–1.90	0.79
DAS28-ESR at week 0	≦ 3.2	1 (reference)				1 (reference)		
	> 3.2	2.62	1.54–4.47		< 0.01^†^	2.53	1.91–3.36	< 0.01^†^
RF positivity*		0.99	0.59–1.65		0.97	1.28	0.95–1.73	0.11
Concomitant use of GC		1.49	0.88	2.51	0.14	1.34	1.01–1.79	0.05^†^
Dose of MTX (mg/week)	0	1 (reference)				1 (reference)		
	1–6	1.02	0.38–2.72		0.97	0.71	0.41–1.22	0.22
	7–9	0.69	0.31–1.57		0.38	0.73	0.47–1.15	0.17
	10–15	0.73	0.34–1.56		0.41	0.63	0.40–0.97	0.04^†^
	> 15	0.48	0.20–1.14		0.10	0.56	0.34–0.91	0.02^†^
Failure in > 2 csDMARDs		1.64	0.67–4.04		0.28	1.09	0.74–1.63	0.66
Biologics	IFX	1 (reference)				1 (reference)		
	ETA	1.35	0.40–4.49		0.63	1.17	0.66–2.09	0.59
	ADA	1.51	0.66–3.44		0.33	0.81	0.55–1.20	0.29
	GLM	3.71	0.67–20.69		0.14	1.55	0.55–4.32	0.41
	CZP	1.13	0.22–5.66		0.89	0.46	0.19–1.12	0.09
Phase	I + II	1 (reference)				1 (reference)		
	III	1.49	0.82–2.70		0.19	0.71	0.52–0.97	0.03^†^

*DAS28* disease activity score for 28 joints, *ESR* erythrocyte sedimentation rate, *MTX* methotrexate, *GC* glucocorticoid, *OR* odds ratio, *CI* confidence interval

*RF > 20 IU/mL was defined as positive

^†^*p* < 0.05

Concomitant use of glucocorticoids seemed to be a common risk for both types of responsiveness. As glucocorticoid use can interact with other variables, logistic regression analysis was conducted including terms of interaction among all of the variables listed in Table 3; however, no significant interactions were observed (data not shown). Therefore, no interaction terms were incorporated in the final analysis. Also, if the glucocorticoid dose had been reduced during TNFi treatment, this could have decreased the erythrocyte sedimentation rate and CRP level and thereby increased the DAS28. Therefore, we calculated the mean difference in the glucocorticoid dose between week 0 and week 22 among the patients taking glucocorticoids (Suppl. Table 1). The mean reductions in the glucocorticoid dose were 0.49 mg/day in the insensitivity group, 0.55 mg/day in the poor response group and 0.35 mg/day in the control group. The glucocorticoid dose was reduced in 8.5% of the TNFi insensitive patients, 11.4% of the poor response patients and 11.8% of the control patients. There was no statistical difference among the three groups.

## Discussion

With advances in the treatment of RA, optimising the outcomes of difficult-to-treat patients has become increasingly important in clinical practice [8, 9]. Although the global consensus is that there is a wide variation in difficult-to-treat statuses, few studies have stratified the patients by response patterns. This cohort study is the first to show an epidemiological difference between insensitivity and a poor response to additive treatment with TNFis in patients with csDMARD-IR and bDMARD-naïve RA. The differences in risk factors detected between these two groups strongly suggest that TNFi insensitivity is not simply a more severe form of a poor response.

Interestingly, the option of infliximab dose escalation in phase III seemed to reduce the risk of a poor treatment response (Table 3 and Supplementary table 1), but no such association was observed in the insensitivity group.

These results may explain the current controversial results on the effectiveness of dose escalation of infliximab for RA patients [23–26]; if patients with TNFi insensitivity are etiologically different from those with a poor response to TNFi, combining these patients into one outcome group as a ‘refractory group’ may mask the net effect of dose escalation among patients with a poor response. Therefore, if a patient responds to TNFi treatment to some extent, it is worth trying to increase the dose, but if disease activity worsens during treatment, increasing the dose is unlikely to have an effect. Further research is needed on whether patients with insensitivity to a TNFi also have a higher risk of insensitivity to other TNFis, to help select the second bDMARD for these patients.

The risk of insensitivity or a poor response also appeared to vary by drug type. Especially, certolizumab showed a higher risk of insensitivity but a lower risk of a poor response, compared with infliximab, although the difference in risk between the two drugs could not be compared due to the small sample size. As this study included only bDMARD-naïve patients, further research is needed to determine the difference in the effectiveness of bDMARDs in different clinical settings.

Our study also suggested that concomitant use of glucocorticoids may modify the responsiveness of bDMARD-naïve patients to TNFis. This is consistent with previous studies that showed glucocorticoids to be a negative predictor of the clinical response to bDMARDs, including TNFis [12, 18] and tocilizumab [27, 28]. Several possible reasons may explain this negative correlation. First, as glucocorticoids mask disease activity, concomitant use of these agents may indicate high disease activity or the presence of refractory disease prior to administration of a TNFi. However, whether this explains the refractory status and/or exacerbation of symptoms during treatment remains uncertain. Second, previous reports suggested that the effect of TNFis is related to monocytes [29, 30]. For example, a TNFi was reported to increase the secretion of the soluble TNF receptor, a natural inhibitor of TNF, and production of interleukin 10 by monocytes [31]. As glucocorticoids reduce the number of peripheral monocytes, the use of glucocorticoids with a TNFi may decrease this monocyte-dependent anti-rheumatic effect of TNFis. Third, changes in the profiles of hypothalamic–pituitary–adrenal axis hormones may have affected the responsiveness to TNFi. Previous studies have shown that endogenous levels of hormones in this axis are closely related to the outcome of TNFi treatment [16], and an increased adrenocorticotropic hormone (ACTH) level, ACTH/cortisol ratio and endogenous cortisol level are predictors of a good outcome [32]. Therefore, it is possible that suppression of ACTH by glucocorticoids may affect the TNFi refractory status.

Even so, transient treatment with low-dose glucocorticoids is reported to be beneficial for preventing bone and joint destruction [33–37] and prolonging life expectancy in patients with RA [38], which makes low-dose glucocorticoids a candidate initial treatment for RA [11]. Therefore, glucocorticoids should not be avoided merely because they may influence the responsiveness to TNFis. Instead, when glucocorticoid-naïve patients are refractory to csDMARDs, the introduction of TNFis before glucocorticoids might be recommended.

This study was limited by its inherently retrospective nature. In particular, the judgement of ‘a lack of response’ was left up to the head physician, which might have resulted in heterogeneity among the patient group with no response to TNFis. Ideally, the judgement of a lack of response should be made by objective data such as the DAS28 at the time of deciding whether to discontinue treatment. However, data at the time of making this decision was available for a limited number of patients only, and we relied on the physician’s judgement for determining a lack of response.

Another limitation is that this analysis could not completely eliminate the effect of background heterogeneity. For example, usage of glucocorticoids may indicate the presence of chronic renal failure, for which administration of high-dose MTX is difficult. In addition to these limitations, there is no global standard for insensitivity to TNFis, and the definition used in this study may need to be validated in future clinical studies. Finally, this research did not elucidate the mechanisms of insensitivity. Further research is needed, including similar studies using other bDMARDs, as well as analyses of the immune profiles of the patients.

## Conclusions

This is the first study to show epidemiological differences between patients with TNFi insensitivity and those with a poor response to TNFis among csDMARD-IR and bDMARD-naïve patients with RA. Differences in the risk factors between the two groups strongly suggest that the two statuses are different entities, although immunological and serological studies are required to elucidate the aetiology. Further research is required including a similar study involving other bDMARDs to increase understanding of the treatment options to avoid incorrect targeting of the disease.

## Supplementary information

**Additional file 1: Table 1.** Dose reduction of glucocorticoid at 22 weeks. Only those who were treated with glucocorticoid are included.

## Data Availability

The datasets generated and/or analysed during the current study are available in the FIRST registry of the University of Occupational and Environmental Health. The datasets are not publicly available due to our privacy policy but are available from the corresponding author on reasonable request.

## Abbreviations

bDMARD: Biologic disease-modifying anti-rheumatic drug
CCP: Citrullinated protein
CRP: C-reactive protein
csDMARD: Conventional synthetic DMARD
csDMARDs-IR: Inadequate response to csDMARDs
DAS28: Disease activity score in 28 joints
HAQ: Health assessment questionnaire
MMP-3: Matrix metalloproteinase-3
MTX: Methotrexate
NTx: Type I collagen cross-linked N-telopeptide
RA: Rheumatoid arthritis
RF: Rheumatoid factor
TNFi: Tumour necrosis factor inhibitor
