# Supplementary material for: Insensitivity versus poor response to tumour necrosis factor inhibitors in rheumatoid arthritis: a retrospective cohort study
Source: Arthritis Res Ther. 2020 Mar 4;22:41. doi: 10.1186/s13075-020-2122-5 (PMC7057565; doi:10.1186/s13075-020-2122-5)
Supplement: Supplementary file 1 — Additional file 1: Table 1. Dose reduction of glucocorticoid at 22 weeks. Only those who were treated with glucocorticoid are included. [file 13075_2020_2122_MOESM1_ESM.docx]

**Supplementary table 1. Dose reduction of glucocorticoid at 22 weeks. Only those who were treated with glucocorticoid are included.**

|  | **TNFi insensitivity** | **Refractory** | **Control** | **p** |
| --- | --- | --- | --- | --- |
| **Mean change in glucocorticoid dose** | -0.49 | -0.55 | -0.35 | 0.24 |
| **% of those whose GC dose was reduced** | 19.05 | 19.88 | 16.91 | 0.51 |
